# Linkage of Emergency Department Patients With Public Benefits Navigators via Text Messages: A Randomized Clinical Trial

**DOI:** 10.1001/jamahealthforum.2025.6637

**Published:** 2026-02-06

**Authors:** Austin S. Kilaru, Aliza Haider, Joseph Harrison, Erica L. Dixon, Lauren Southwick, Melissa Berkowitz, Charles Rareshide, Conor Carroll, Clayton Kaledin, Grace McDermott, Michael Mehta, Alisa J. Stephens Shields, Wendy De La Rosa, Anish K. Agarwal, Raina M. Merchant

**Affiliations:** 1 Center for Healthcare Transformation and Innovation, Penn Medicine, Philadelphia, Pennsylvania; 2 Department of Emergency Medicine, Perelman School of Medicine, University of Pennsylvania, Philadelphia; 3 Editorial Fellow, *JAMA Health Forum*; 4 Georgetown University School of Medicine, Washington, DC; 5 San Francisco Veteran’s Affairs Medical Center, San Francisco, California; 6 Department of Medical Ethics and Health Policy, University of Pennsylvania, Philadelphia; 7 Center for Health Incentives and Behavioral Economics, University of Pennsylvania, Philadelphia; 8 Benefits Data Trust, Philadelphia, Pennsylvania; 9 Sellers Dorsey, Philadelphia, Pennsylvania; 10 Clarifi, Philadelphia, Pennsylvania; 11 Case Western Reserve University School of Medicine, Cleveland, Ohio; 12 Department of Biostatistics, Epidemiology and Informatics, Perelman School of Medicine, University of Pennsylvania, Philadelphia; 13 The Wharton School, University of Pennsylvania, Philadelphia

## Abstract

**Importance:**

Enrollment in public benefits is associated with improved health outcomes, yet many eligible individuals do not claim them. Public benefit programs are important policy tools to address health-related social needs. Health systems have developed new partnerships with community organizations to assist patients with enrollment in benefits.

**Objective:**

To determine whether text messages were more effective than paper referrals in prompting patients to contact benefits navigators following discharge from the emergency department.

**Design, Setting, and Participants:**

This 2-arm, nonblinded, randomized clinical trial was conducted from November 2023 to April 2024 at 2 academic hospital emergency departments in Philadelphia, Pennsylvania, and included adult individuals in stable condition with Medicaid or Medicare insurance who were discharged from the emergency department and eligible for at least 1 of 10 public benefit programs. Individuals were excluded if they were unable to read English or did not have access to a mobile phone. Data were analyzed from May 2024 to November 2024.

**Interventions:**

Eligible participants were randomized in a 1:1 ratio. Participants allocated to the intervention received a series of 4 automated text messages over 14 days that prompted them to contact a benefits navigator telephone line operated by a community partner; those allocated to the control group received a paper flyer.

**Main Outcomes and Measures:**

The primary outcome was whether study participants called benefits navigators within 14 days. Secondary outcomes included whether study participants submitted any benefits application within 14 days.

**Results:**

Of 1778 patients screened, there were 160 participants enrolled. Participants’ mean (SD) age was 44 (17) years; 94 (59%) were women, 145 (91%) were non-Hispanic Black, and 11 (7%) were non-Hispanic White. In the intervention group, 20 participants (25%) contacted benefits navigators vs 0 in the control group (difference, 25 percentage points; 95% CI, 16%-35%). In the intervention group, 11 participants (14%) submitted at least 1 application for public benefits compared with 0 in the control group (difference, 14 percentage points; 95% CI, 6-22).

**Conclusions and Relevance:**

The trial results suggest that text messages were more effective than paper referrals to help eligible emergency department patients seek assistance with public benefits applications. Text messages may offer a tool to allow health systems, in collaboration with community partners, to address health-related social needs.

**Trial Registration:**

ClinicalTrials.gov Identifier: NCT05654220

## Key Points

**Question:**

Are text messages more effective than paper referrals in encouraging emergency department patients to seek assistance in applying for unclaimed public benefits?

**Findings:**

In this randomized clinical trial of 160 patients who were discharged from the emergency department, 25% who received text message reminders contacted benefits navigators compared with 0 who received paper flyers alone.

**Meaning:**

The trial results suggest that health system interventions that address social needs, including benefits navigation, may benefit from using text messages in their design.

## Teaser

This randomized clinical trial examines whether text messages were more effective than paper referrals in prompting patients to contact benefits navigators following discharge from the emergency department.

## Introduction

In the US, public benefit programs are an important policy mechanism to address health-related social needs (HRSN), providing financial assistance, food access, housing support, and access to health care.^1,2,3,4^ Enrollment in public benefits is associated with improved physical and mental health outcomes.^5,6,7,8,9,10,11,12,13,14^ However, tens of billions of dollars available through federal, state, and municipal public benefits are unclaimed every year.^15,16,17,18,19^ For example, it has been estimated that fewer than 1 in 5 eligible households receive financial assistance for energy bills through the Low Income Home Energy Assistance Program (LIHEAP).^20^

Screening patients for HRSN that affect clinical outcomes is an emerging priority for health care organizations.^21,22,23^ Health system interventions that address HRSN have proliferated,^24,25,26,27,28^ including partnerships between health care and community organizations that screen and assist patients with enrollment in public benefits.^29,30,31,32^ These interventions have primarily occurred in the outpatient primary care setting. However, individuals who do not regularly engage with primary care may seek services in emergency departments (EDs).^33,34,35,36,37^ While EDs serve an essential role in the health care safety net, they do not commonly offer assistance in addressing long-term social needs.^38^

We report results from the Linking Emergency Department Patients to Assistance Programs study, a randomized clinical trial that evaluated whether text messages could encourage patients to apply for public benefits following discharge from the ED. Text message interventions have been tested in various settings to improve health care delivery but less commonly to address HRSN.^39,40,41,42,43,44,45^ Our goal was to determine whether text messages facilitated engagement with public benefits navigators more effectively than referral via paper flyer, a tool commonly used to distribute resources to patients.

## Methods

### Study Design and Setting

We conducted a 2-arm, nonblinded, prospective randomized clinical trial between November 2023 and April 2024 in 2 academic hospitals located in Philadelphia, Pennsylvania (Supplement 1). Data were analyzed from May 2024 to November 2024. This study was conducted in partnership with Benefits Data Trust (BDT), a nonprofit organization that operated a telephone hotline staffed by trained public benefits navigators. BDT and the research team signed a partnership agreement that involved the creation of a dedicated BDT phone number, periodic reporting of call volume and applications submitted, and a data sharing agreement. The institutional review board at the University of Pennsylvania approved this study, and participants provided written informed consent. We followed the Consolidated Standards of Reporting Trials (CONSORT) reporting guidelines for reporting randomized clinical trials.^46^ The study was registered on ClinicalTrials.gov (NCT05654220).

### Study Participants

We recruited participants from patients who were undergoing evaluation and treatment in 2 EDs (eFigure 1 in Supplement 2). Trained research staff used the electronic health record to screen adult ED patients for inclusion criteria, including an emergency severity index triage score of at least 3 (indicating a lower likelihood of severe illness) and active Medicaid or Medicare insurance. Research staff approached ED clinicians to assess exclusion criteria, including whether patients had medical instability, were unable to communicate, in police custody, had alcohol intoxication, or were likely to be hospitalized. Research staff then screened potential participants to confirm eligibility, including the ability to read English, access to a mobile phone, and residence in Philadelphia, Pennsylvania. Participants could be approached for recruitment at any moment during their clinical encounter between ED triage and discharge, with research staff identifying appropriate moments between clinical activities. Patients in these EDs received routine screening for concerns, including domestic violence, depression, and substance use disorder, but were not screened for benefits eligibility outside of the context of this study.

Participants completed an online screening tool, the Benefits Launch Express, which was designed for Philadelphia residents to assess enrollment and eligibility for public benefits, and were compensated $20. While Benefits Launch Express was proprietary to BDT, similar screening tools, such as Benefits.gov, BenefitsCheckUp.org, and state-specific portals, can provide similar screening information. Participants had to be eligible for and not currently enrolled in at least 1 of 10 benefit programs for which BDT provided direct application assistance (eTable 1 in Supplement 2). These benefits included LIHEAP, the Property Tax and Rent Rebate Program, Supplemental Nutrition Assistance Program (SNAP), homestead exemption, and the senior food box program. Two benefits, the Child Care Income Subsidy and Children’s Health Insurance Program (CHIP), applied to participants with minor dependents. Three benefits applied to participants with Medicare: the Pharmaceutical Assistance Contract for the Elderly Program, Medicare Savings Program, and the low-income subsidy. BDT provided help applying for 10 additional benefits, including the earned income tax credit and child tax credit, although eligibility for these programs alone did not qualify for study participation.

### Study Interventions

Eligible participants were randomly allocated 1:1 to the 2 study groups in block sizes of 6 (research staff were masked to block size). All participants were informed of the benefits for which they were potentially eligible and given a paper flyer that included the BDT phone number (eFigure 3 in Supplement 2). Intervention participants then received a series of automated text messages sent via Way to Health (University of Pennsylvania), a technology platform that delivers text messages for clinical and research purposes directly to participants’ mobile phones.^47^ Participants received messages on days 1, 3, 7, and 14 following discharge, with adjustments to ensure that messages were received during weekday business hours (eTable 2 in Supplement 2). Text messages included prompts to call BDT and the phone number. The content (eFigure 2 in Supplement 2) incorporated behavioral strategies with previously demonstrated effectiveness in text message interventions, including loss aversion, psychological ownership, use of budgeting periods, and social norming.^48,49,50^ All participants received a text message notification at 14 days that the study had ended, which was followed by a brief survey. At any time, participants could discontinue receipt of messages and study enrollment by responding “BYE.” The study team monitored message transmission and retransmitted if the initial message failed to send.

### Outcomes

The primary outcome was a call to BDT within 14 days to seek assistance with public benefits applications. To measure this outcome, we designated a specific telephone line and obtained data from BDT for all individuals who called and spoke to a benefits navigator. The study team reviewed additional call data to determine whether participants called BDT using other telephone lines or whether other individuals (eg, family members) called on behalf of the participant. Secondary outcomes included whether study participants applied for any public benefits within 14 days. We also assessed these outcomes at 30 days.

All participants completed a baseline survey on enrollment to provide additional data on demographic characteristics, social needs, and prior experiences with public benefits (eMethods 1 in Supplement 2). Survey questions on social needs were adapted from the Accountable Health Communities Screening Tool.^21^ To further describe patient characteristics, we also obtained data from electronic health records. Regarding insurance type, patients who were dually enrolled in Medicaid and Medicare were categorized in the Medicare group due to their eligibility for Medicare-specific benefits.

### Statistical Analysis

The trial was designed to provide 80% power using a 1-sided significance level of .05 to detect a difference for the primary outcome, with a 10% success rate in the treatment group and 1% success rate in the control group. We estimated these rates based on pilot testing before the trial. Given that we were primarily interested in whether text messages were more effective than paper flyers (which represented the standard of care), we elected to use 1-sided testing to optimize study efficiency (eMethods 2 in Supplement 2).

In the primary analysis, we used an intent-to-treat approach. We retained participants to the arm to which they were randomly assigned, regardless of whether patients discontinued receipt of text messages. We used descriptive statistics to summarize differences in patient characteristics between groups. For primary and secondary outcomes, we compared outcomes between groups using 1-sided *z* tests for proportions and reported differences in proportions with 1-sided 95% CIs. To account for residual imbalances in baseline benefits eligibility between study arms, we conducted post hoc sensitivity analyses using multivariable logistic regression models for primary and secondary outcomes. These models used the Firth penalized likelihood approach to account for the rarity of the outcome. Statistical tests were considered significant at α < .05. Analyses were performed with SAS, version 9.4 (SAS Institute).

## Results

### Study Population

**Figure 1.**
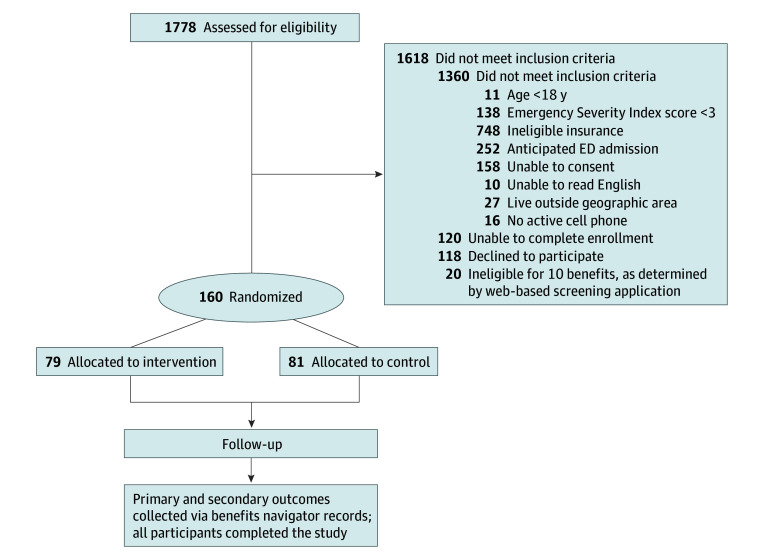
Consort Diagram ED indicates emergency department.

Of 1778 screened, 160 patients (0.9%) were deemed eligible and randomly assigned (Figure 1). Of patients that were excluded, 1360 patients did not meet inclusion criteria, 120 were unable to complete enrollment due to changes in condition or interruptions by clinical activities, and 118 patients declined to participate. There were also 20 patients who completed the online screening tool and were not eligible for any relevant benefits.

An error occurred in the allocation of 1 participant that resulted in an imbalance of participants assigned to the intervention (79 [49%]) and control (81 [51%]) groups. One participant allocated to the intervention group elected to discontinue receipt of text messages during the study, and 10 intervention participants(13%) did not receive the full set of text messages, although all participants received at least 1 message (eTable 3 in Supplement 2).

The mean (SD) age for participants was 44 (17) years; 94 (59%) were women and 60 (38%) were men. There were 145 patients (91%) who self-reported non-Hispanic Black race and ethnicity, 11 (7%) were non-Hispanic White, and 4 (3%) self-reported other race and ethnicity or declined to provide information. Most patients had Medicaid insurance (131 [82%]), and the remaining had Medicare (28 [18%]). A total of 114 patients (71%) reported that they previously applied for at least 1 public benefit program. Additional participant characteristics are described in Table 1 (eTables 4-6 in Supplement 2).

**Table 1.** Participant Characteristics by Study Arm

Characteristic	No. (%)		
	All patients (N = 160)	Intervention (n = 79)	Control (n = 81)
Age, mean (SD), y	44 (17)	44 (16)	43 (17)
Sex			
Female	94 (59)	45 (57)	49 (60)
Male	60 (38)	30 (38)	30 (37)
Self-reported other/decline to answer	6 (4)	4 (5)	2 (2)
Race and ethnicity			
Non-Hispanic Black	145 (91)	71 (90)	74 (91)
Non-Hispanic White	11 (7)	8 (10)	3 (4)
Self-reported other	4 (3)	0	4 (5)
Health insurance			
Medicaid	131 (82)	65 (82)	66 (81)
Medicare	29 (18)	14 (18)	15 (19)
People living in household			
1	39 (24)	21 (27	18 (22)
2	46 (29)	21 (27)	25 (31)
3	33 (21)	20 (25)	13 (16)
4	22 (14)	10 (13)	12 (15)
≥5	20 (13)	7 (9)	13 (16)
Annual household income, $			
<20 000	83 (52)	44 (56)	39 (48)
20 000-39 000	31 (19)	11 (14)	20 (25)
>40 000	20 (13)	12 (16)	8 (9)
Declined to answer	26 (16)	12 (15)	14 (17)
Education level			
Less than high school	21 (13)	9 (11)	12 (15)
High school diploma	84 (53)	46 (58)	38 (47)
Some college or higher	54 (34)	24 (30)	30 (37)
Declined to answer	1 (1)	0	1 (1)
Previous application for public benefits			
Yes	114 (71)	53 (67)	61 (75)
No	43 (27)	23 (29)	20 (25)
Decline to answer	3 (2)	3 (4)	0
Previous barriers to public benefits applications			
None	117 (74)	56 (71)	62 (77)
Uncertain how to apply	19 (12)	12 (15)	7 (9)
Too confusing or overwhelming	6 (4)	3 (4)	3 (4)
No permanent address	5 (3)	3 (4)	2 (2)
Unable to collect paperwork	4 (3)	2 (3)	2 (2)
Application took too long to complete	3 (2)	0	3 (4)
Did not want to give information to the government	2 (1)	1 (1)	1 (1)
Not a person who would ever apply for benefits	2 (1)	1 (1)	1 (1)
Concerned about what others would think	0	0	0

Among all study participants, the most frequent eligible benefit was LIHEAP (124 [78%]), followed by the Property Tax and Rent Rebate Program (53 [33%]), CHIP (52 [33%]), and SNAP (33 [21%]; Table 2). More patients allocated to the intervention group were eligible for SNAP (24 [30%]) than in the control group (9 [11%]). However, more patients in the control group were eligible for LIHEAP (66 [81%]) and CHIP (31 [38%]) than in the intervention group (58 [73%] and 21 [27%], respectively).

**Table 2.** Public Benefits Eligibility for Participant or Household Member by Study Arm as Determined by Initial Screening

Benefit	Type	No. (%)		
		All patients (N = 160)	Intervention (n = 79)	Control (n = 81)
Low Income Home Energy Assistance Program	Federal	124 (78)	58 (73)	66 (81)
Property Tax & Rent Rebate Program	State	53 (33)	27 (34)	26 (32)
Children’s Health Insurance Program	State	52 (33)	21 (27)	31 (38)
Supplemental Nutrition Assistance Program	Federal	33 (21)	24 (30)	9 (11)
Child care income subsidy	State	32 (20)	13 (16)	19 (23)
Homestead exemption	Municipal	20 (13)	9 (11)	11 (14)
Senior food box program	Federal	1 (1)	0	1 (1)
Available only to participants with Medicare				
No.	NA	29	14	15
Pharmaceutical Assistance Contract for the Elderly Program	State	8 (29)	5 (38)	3 (20)
Medicare Savings Programs	State	6 (21)	3 (23)	3 (20)
Low-income subsidy	Federal	3 (11)	1 (8)	2 (13)

Abbreviation: NA, not applicable.

### Study Outcomes

In the intervention group, 20 participants (25%) called BDT within 14 days vs 0 participants in the control group (difference, 25 percentage points [pp]; 1-sided 95% CI, >17). At 30 days, 24 participants (30%) called BDT compared with 2 (2%) in the control group (difference, 28 pp; 1-sided 95% CI, >19; Table 3).

**Table 3.** Primary and Secondary Study Outcomes

Outcome	No. (%)		Difference, pp (1-sided 95% CI)	*P* value
	Intervention (n = 79)	Control (n = 81)		
Call to benefits navigator within 14 d	20 (25)	0	25 (>17)	<.001
Call to benefits navigator within 30 d	24 (30)	2 (2)	28 (>19)	<.001
Any benefits application submitted within 14 d	11 (14)	0	14 (>7)	<.001
Any benefits application submitted within 30 d	14 (18)	0	18 (>11)	<.001
Total No. of applications submitted within 14 d	14	0		
Supplemental Nutrition Assistance Program	4 (29)	NA	NA	NA
Property Tax & Rent Rebate Program	4 (29)			
Low Income Home Energy Assistance Program	3 (21)			
Senior food box program	3 (21)			
Unemployment insurance	2 (14)			
Social security disability insurance	1 (7)			
Medicaid	1 (7)			
Total No. of applications submitted within 30 d	23	0		
Supplemental Nutrition Assistance Program	6 (26)	NA	NA	NA
Property Tax & Rent Rebate Program	5 (22)			
Low Income Home Energy Assistance Program	4 (17)			
Senior food box program	3 (13)			
Unemployment insurance	2 (9)			
Social security disability insurance	1 (4)			
Medicaid	1 (4)			
Earned income tax credit	1 (4)			

Abbreviations: NA, not applicable; pp, percentage point.

In the intervention group, 11 unique participants (14%) submitted at least 1 application for public benefits within 14 days, compared with 0 in the control group (difference, 14 pp; 1-sided 95% CI, >7). At 30 days, 14 unique participants (18%) submitted at least 1 application, compared with 0 in the control group (difference, 18 pp; 1-sided 95% CI, >11). Among participants in the intervention group, there were 14 distinct applications submitted within 14 days and 23 distinct applications submitted within 30 days. Sensitivity analyses that adjusted for baseline differences between groups demonstrated similar results (eTable 7 in Supplement 2).

**Figure 2.**
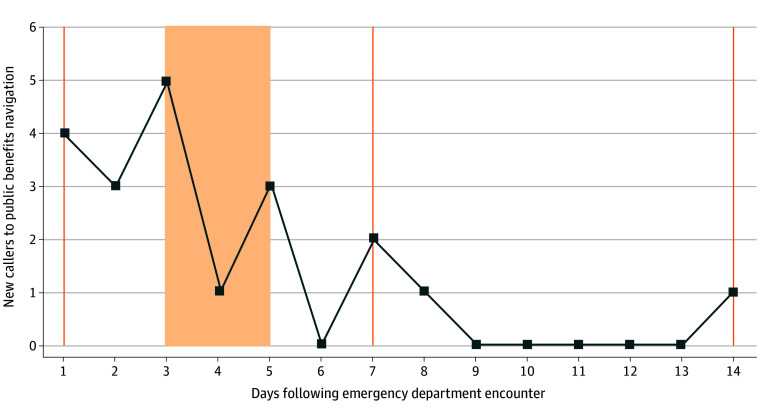
Timing of Calls to Public Benefits Navigator Following Emergency Department Encounter Results for intervention group shown only. Text messages sent on days are indicated by orange vertical lines and include days 1, 3, 7, and 14, with day 1 and day 3 messages adjusted to the next business day if occurring during the weekend. See eTable 2 of Supplement 2 for the text message schedule as listed by week day of study enrollment. The shaded area indicates the days over which patients might receive the day 3 message.

We examined the frequency of calls during the 14-day intervention period for participants receiving text messages (Figure 2). Most calls occurred within the first 3 days after discharge, with the highest number of calls occurring on days that patients received text messages (days 1 and 3). Only two calls were made after day 7 during the 14-day intervention period.

## Discussion

In this randomized clinical trial, ED patients randomized to receive either text messages or a paper flyer that prompted them to seek assistance to apply for public benefits. While no participants who received the flyer called benefits navigators, 1 in 4 participants who received text messages did call. Of those that called, more than half submitted a benefits application. These findings suggest that text messages may offer a strategy to assist patients with HRSN, even if that intervention is not directly related to the reasons for their health care encounter.

The participation gap between eligibility and enrollment for public benefit programs is well established and has been attributed to complicated and shifting eligibility criteria, as well as difficulty navigating the enrollment process.^15,16,20,51^ Several policy proposals have attempted to close this gap. In 2023, the US General Services Administration launched pilot initiatives that use text message notifications to promote enrollment in federally funded benefits.^52^ In combination with mail and telephone notifications, text messages have improved timely benefit renewals among patients enrolled in Medicaid and SNAP. Our study is notable in that we used text messages in a novel context among individuals who were identified not because of prior benefits applications or enrollment, but who were instead screened while receiving ED care. The text message intervention is generalizable to other communities, many of which already offer screening tools and service centers that can assist with public benefits applications.

There is broad consensus that poverty is a major driver of health outcomes and that increased access to social services positively affects health outcomes.^53,54,55^ Accordingly, health care organizations have increased screening for patients for HRSN^22,56^ but often cannot directly address those needs, requiring linkage to community-based services.^57^ Health care organizations have developed cross-sector collaborations with social services organizations to help individuals with housing insecurity, lack of transportation, and unsafe neighborhood or interpersonal conditions.^58^ Partnerships for benefits navigation expand those interventions.^31,59^ While these partnerships have potential to improve economic stability, the optimal design and implementation of these interventions remains unclear, and there is limited evidence on the effect of these programs on either financial or physical health.^58^ In this context, our study has 3 implications.

First, text messages delivered over a 2-week follow-up period were more effective at promoting engagement than distribution of a paper flyer. Referrals and social resources are still commonly delivered by paper to patients who are discharged from clinical care, often packaged with discharge instructions. Prior studies have examined the use of text messaging following ED discharge to promote medication adherence, monitor symptoms, or improve attendance at outpatient follow-up visits.^42,60,61,62^ Text messaging has also been tested as an intervention to promote healthy behaviors, including physical activity, weight loss, and reduced substance use.^45,63^ While developing text-based interventions for research purposes may incur high start-up costs, the cost of text message applications is lower when they can be scaled across health system operations as part of clinical care. However, to our knowledge there is limited evidence on the use of text messaging for HRSN interventions in the ED or hospital setting.

In the control group, the absence of any response to the flyer was striking. To participate in this study, even patients in the control group were screened and informed of their eligibility for public benefits. We hypothesize that text messages were more effective because they provided ongoing engagement following the initial discussion, which occurred in the ED setting when patients were likely focused on obtaining medical evaluation and treatment. Further support for this hypothesis is suggested by the pattern of calls observed in this trial, which generally occurred on the same day that messages were sent. The 2 participants who did call in the control group did so after receiving a follow-up survey on day 14, which was delivered via text message.

The second implication is that at least some ED patients were willing to discuss and address financial needs, even if these needs were unrelated to the reason for their health care encounter. Not all patients were willing to engage with study personnel. After applying eligibility criteria to exclude patients with severe illness or incapacitation, 40% agreed to participate, with the remainder declining or unable to do so due to clinical interruptions. These findings suggest that the intervention was not appropriate for all ED patients, but that there are some who may welcome the engagement while awaiting care.

Screening for HRSN has become more common in health care settings but remains uncommon in the ED, setting apart from concerns requiring urgent intervention, such as frequent falls, substance use disorder, or depression.^64,65^ Patients may be asked about food, housing, and transportation needs by ED clinicians, including nurses and social workers, but it is less common for individuals to receive determination of eligibility for benefits. Therefore, the intervention under evaluation in this study, which both study arms received, included in-depth screening for public benefits that represented a departure from usual practice in the ED setting. An important question is whether ED patients would respond to text messages even without in-person screening and engagement. An alternative, and potentially more scalable, study design might contact all potentially eligible patients who were discharged from the ED with automated text messages.

The third implication is that benefits navigation can be implemented in nonlongitudinal care settings, such as the ED or hospital. These partnerships most commonly occur in primary care settings.^31,59^ Primary care may be the optimal setting for colocated benefits interventions, given that most patients do not have acute illness and have trusted relationships with clinicians. The ED is a more unpredictable environment to introduce these services; however, there are advantages to expanding beyond primary care to reach individuals who may not have a regular source of care.

### Limitations

This study had several limitations. We excluded patients who did not speak English, patients with no active insurance due to concern for competing Medicaid applications from financial counselors from the health system (and for whom public benefits assistance may be particularly valuable), and patients with commercial insurance, even though they may have been eligible for benefits. Baseline eligibility for specific benefits was unequally distributed across study arms despite randomization. Although adjusted analyses accounted for these differences, we could not control for unobserved confounders. We were also unable to capture outcomes for participants that may have called BDT but ended the call before providing their information, and we may not have captured some callers if participants asked friends or family to call BDT for them. Due to state-based data protection policies, we also could not access information regarding whether benefits applications were successful and whether benefits were used; however, this would be an important expansion for future work. Another limitation was that research personnel who recruited participants were not masked to treatment allocation due to the feasibility of setting up the technology to ensure that participants received messages; this lack of masking may have biased more participants in the treatment group to respond favorably to text messages. Finally, this study was conducted during a period when COVID-19 pandemic–era policies for automated renewals for certain benefits, such as Medicaid and SNAP, were discontinued, leading to potential uncertainty over benefits eligibility and affecting study participation, although these effects likely affected participants in both study arms.^66^

## Conclusions

This randomized clinical trial screened ED patients for eligibility for unclaimed public benefits and found that text messages were more effective than paper flyers at prompting patients to seek assistance with public benefits applications following discharge. Text messages may be incorporated into efforts to address individual health-related social needs, even in health care settings that do not typically offer these interventions.

## Notes

Supplement 1. Trial protocol

Supplement 2. eFigure 1. Study overview diagram eTable 1. List of public benefits with application support offered by benefits navigators from Benefits Data Trust eTable 2. Schedule of text messages according to weekday of study enrollment eFigure 2. Automated text messages used in the intervention arm eFigure 3. Flyer distributed to control and intervention groups eMethods 1. Baseline survey eMethods 2. Power analysis eTable 3. Frequency of discontinued and failed text messages in the intervention group eTable 4. Baseline survey—health-related social needs eTable 5. Baseline survey—previous experience with public benefits applications eTable 6. Final survey responses eTable 7. Sensitivity analysis (adjusted model)

Supplement 3. Data sharing statement
