# Supplementary material for: Linkage of Emergency Department Patients With Public Benefits Navigators via Text Messages: A Randomized Clinical Trial
Source: JAMA Health Forum. 2026 Feb 6;7(2):e256637. doi: 10.1001/jamahealthforum.2025.6637 (PMC12881984; doi:10.1001/jamahealthforum.2025.6637)
Supplement: Supplement 1. — Trial protocol [file jamahealthforum-e256637-s001.pdf]

Final Trial Protocol
Linking Emergency department patients to Assistance Programs (LEAP) study
Version – 1/12/2024

PRINCIPAL INVESTIGATOR: Austin Kilaru, MD, MSHP

PROTOCOL TITLE: A randomized controlled trial to link emergency department patients to navigation services for enrollment in public benefits: the LEAP study

OBJECTIVES:

The objective of this randomized controlled trial is to test the effect of screening patients in Penn Medicine Emergency Departments for eligibility of public benefits programs and using text messages post-discharge to connect patients to benefits enrollment specialists at Benefits Data Trust (BDT). Eligible patients will be randomly selected to receive text messages for two weeks after Emergency Department discharge with the phone number to speak with a benefits enrollment specialist at BDT. The number of calls to the BDT phone line and the number of submitted applications to public benefits programs will be compared between patients receiving a summary flyer with the phone number for BDT and the text message intervention to connect with BDT in comparison to an active control group who receives only a summary flyer with the phone number for BDT.

Primary outcome variable(s): Participant connection to BDT measured by phone calls completed by confirmed participants to BDT.

Secondary outcome variable(s): Number of applications for public benefits programs submitted per participant by BDT.

BACKGROUND:

Every year, Philadelphians fail to claim approximately $450 million dollars in federal and state benefits[1]. Across the United States unclaimed benefits are estimated at approximately $60 billion[1]. These benefits include support for food, housing, healthcare, economic support, and others that can make a significant impact on well-being, upward mobility, and financial stability. The reasons that benefits are unclaimed are numerous, including lack of awareness about benefit programs and eligibility criteria, lack of agency and self-efficacy in completing expansive application requirements and organizing the necessary paperwork, and navigating the psychological costs of stigma associated with seeking public benefits[2]. In accordance with incentive structures developed as a result of the Affordable Care Act, health care systems have demonstrated value in screening for patients' social needs and creating partnerships with public service agencies to connect patients to social services to improve individual and population health and reduce health care disparities[3-5]. Patients presenting to emergency departments are more likely to possess unmet social needs[6-8]. Thus, it is critical for health care providers in emergency departments to effectively identify patients' social needs and connect patients to social services agencies that can provide both immediate and long-term assistance through the connection to public benefits programs.

Benefits Data Trust (BDT), a benefits support organization based in Philadelphia, has a long track record of success in consistently securing benefits for individuals with unmet social needs. BDT assists individuals with the completion and submission of applications for 19 public benefits programs (e.g., Supplemental Nutrition Assistance Program, the Low-Income Home Energy Assistance Program, and the Pharmaceutical Contract for the Elderly) via different channels, including web, phone, text, and in-person support services. Trained outreach specialists are knowledgeable about the intricacies of benefits applications and eligibility requirements, and support is available in multiple languages. Building trust with clients and serving vulnerable underserved communities is a central focus for outreach specialists. For

services that they do not provide assistance for, BDT is also staffed to provide warm handoffs to help connect individuals to relevant organizations.

The focus of this proposal is to test whether patients identified in Penn Medicine Emergency Departments (ED) randomized to receive a warm handoff text messaging intervention are more likely to connect to study-specific BDT phone line and submit more applications for public benefits programs in comparison to patients who only receive a summary flyer with the BDT study-specific phone line upon discharge from the Emergency Department.

We propose to first survey patients to determine their eligibility for public benefits programs. Second, patients who are eligible for at least 1 of the 10 benefits programs to which BDT provides direct application support to complete applications will be randomly selected to either receive a flyer with the study-specific BDT phone number to apply for public benefits programs (active control) or receive a text messaging intervention for two weeks after leaving the ED with instructions to connect to the BDT study-specific phone line in addition to the flyer. Patients randomized to the intervention arm will receive an initial text message one day post-discharge from the ED with instructions for connecting with BDT and will receive subsequent reminders to connect with BDT on Days 3, 7, and 14 post-discharge. Patients who indicate that they have successfully connected with BDT on Day 3 will not receive intervention messages on Days 7 and 14. All patients, both those randomized to the control and intervention groups, will receive an end-of-study survey on Day 14 post-discharge from the ED to assess the patient's experience in the research study and provide a reminder about the number to connect with BDT.

Insights from this randomized controlled trial will inform future work evaluating benefits enrollment outcomes, the relationship between benefits enrollment and health care outcomes, and variations in health services use among patients connected to BDT in the ED.

References

1. Thompson N. Philadelphians leave $450 million on the table in federal and state benefits annually. *Al Dia News.* March 21, 2021. https://aldianews.com/en/leadership/advocacy/get-your-benefits
2. Barnes CY. "It Takes a While to Get Used to": The Costs of Redeeming Public Benefits. *Journal of Public Administration Research and Theory*. 2021; 31(2), 295-310. https://10.1093/jopart/muaa042
3. Berkowitz SA, Palakshappa D, Rigdon J, Seligman HK, & Basu S. Supplemental Nutrition Assistance Program Participation and Health Care Use in Older Adults. *Ann Intern Med.* 2021; 174(12), 1674-1682. https://10.7326/M21-1588
4. Kreuter MW, Thompson T, McQueen A, & Garg R. Addressing Social Needs in Health Care Settings: Evidence, Challenges, and Opportunities for Public Health. *Annual review of public health*. 2021; 42, 329–344. https://doi.org/10.1146/annurev-publhealth-090419-102204
5. VonHoltz LA, Murray AL, & Cullen DL. Family Connects: A Novel Social Needs Program Within a Pediatric Emergency Department. *Academic pediatrics*. 2022; S1876-2859(22)00150-4. Advance online publication. https://doi.org/10.1016/j.acap.2022.03.002
6. Doran KM, Johns E, Zuiderveen S, Shinn M, Dinan K, Schretzman M, Gelberg L, Culhane D, Shelley D, & Mijanovich T. Development of a homelessness risk screening tool for emergency department patients. *Health Services Research*. 2022; 57(2), 285-293. https://https://doi.org/10.1111/1475-6773.13886
7. Malecha PW, Williams JH, Kunzler NM, Goldfrank LR, Alter HJ, & Doran KM. Material Needs of Emergency Department Patients: A Systematic Review. *Academic emergency medicine : official journal of the Society for Academic Emergency Medicine*. 2018; 25(3), 330–359. https://doi.org/10.1111/acem.13370
8. Samuels-Kalow ME, Boggs KM, Cash RE, Herrington R, Mick NW, Rutman MS, Venkatesh AK, Zabbo CP, Sullivan AF, Hasegawa K, Zachrison KS, & Camargo Jr CA. Screening for Health-Related Social Needs of Emergency Department Patients. *Annals of emergency medicine.* 2021; 77(1), 62–68. https://doi.org/10.1016/j.annemergmed.2020.08.010

CHARACTERISTICS OF THE STUDY POPULATION:

1. Target Population and Accrual:

The target population of this study includes patients presenting in Penn Medicine Emergency Departments (ED).

Target sample size: 188 patients
Pilot study: 30 patients
RCT: 158 patients

Power calculations were based on preliminary testing and a prior assumptions that a baseline rate of about 1% of patients would contact BDT after receiving a flyer at the time of discharge (control group), and 10% of patients would contact BDT after receiving text messages (intervention group). Based on these parameters, we estimate that a sample of 158 patients (79 per arm) would provide at least 80% power to detect a 9% difference between the two study arms, with one-sided statistical testing.

For practical reasons related to study funding and allocation of research staff, we may include one pre-specified interim analysis.

Alpha spending function using O'Brien Fleming efficacy boundary

For k = 2		
P	0.0088	0.0467
N	80	160

2. Key Inclusion Criteria:

Patients will be included in the study based on the following criteria:

- Adults at least 18 years of age
- Patients must legally reside in the city of Philadelphia
- Patients receiving care from Penn Medicine Emergency Departments at the Hospital of the University of Pennsylvania and Penn Presbyterian Medical Center who are deemed unlikely to require inpatient hospitalization or observation at the time of enrollment
- Patients must have one of the following insurance plans: Medicaid and/or Medicare (managed and traditional).
- Patient must be able to read/write in English.
- Patients must be eligible for at least 1 of 10 the benefits programs for which BDT can provide either direct application assistance or provide referral to the social services agency website to submit applications.

3. Key Exclusion Criteria:

Patients will be excluded from participating in the study based on the following criteria:

- Individuals < 18 years
- Does not speak/read English
- Unable to consent or communicate
  - Patients deemed by ED physicians to be in critical or unstable condition
  - Patient is in severe distress, e.g., respiratory, physical, or emotional distress
  - Patient is intoxicated, unconscious, or unable to appropriately respond to questions
- Patients under police custody
- Patients without a stable mobile phone number for the next 21 days

4. Subject Recruitment and Screening:

Patients will be recruited in Penn Medicine Emergency Departments (EDs) at the Hospital of the University of Pennsylvania and Penn Presbyterian Medical Center by trained clinical research assistants. Clinical research assistants will pre-screen patients in the ED via the Electronic Health Record (EHR) to determine whom to approach. Patients will be considered appropriate to approach if they meet the inclusion and exclusion criteria specified above and are determined to be likely discharged from the ED. Patients potential to be triaged to be discharged from the ED will be identified using the following recruitment guidelines: 1) First, patients should have a low acuity level as indicated by the Emergency Severity Index (ESI) level in the EHR. Acuity levels range from Level 1 (highest acuity) to Level 5 (lowest acuity). Patients at levels 3-5 will be prioritized. 2) Clinical research staff will then approach clinician staff in charge of patient care (e.g., physicians or nurses) to confirm the probability of the patient's discharge from the ED.

Eligible patients will be approached in the ED, provided with a brief introduction to the study, and asked if they are interested in participating. Interested participants will complete a brief eligibility survey that confirms the final inclusion and exclusion criteria for eligibility. Participants who are deemed initially eligible will then provide written electronic informed consent in REDCap followed by the Benefits Launch Express (BLX) survey. The BLX survey is a web-based survey managed by BDT which provides initial information about a participant's potential eligibility for at least one of the benefits programs for which BDT provides direct application support or referral. Completion of the BLX survey is necessary to confirm final eligibility for the study. Results of the BLX survey will be manually transferred from the BLX webpage back into the REDCap eligibility survey. After completion of the BLX survey, eligibile and interested participants will create an account in the Way To Health platform, a brief baseline survey, and then proceed to be randomized. All communications with the patients will express that participation is completely voluntary and that they will be free to stop participating at any time.

We will use the Academic Associate (AA) Program at the Emergency Departments (ED) at the Hospital of the University of Pennsylvania and Penn Presbyterian Medical Center to assist with identifying patients. The AA Program provides pre-medical and pre-health post-baccalaureate students an opportunity to engage in clinical and translational research activities. The AAs have Immunization records (measles, mumps and rubella [MMR] and varicella [chickenpox]) and TB screening (PPD test or equivalent), and influenza (in season) and complete a web-based course sponsored by the University of Pennsylvania on the protection of human subjects in research. Using Epic tools, AAs, and trained clinical research coordinators, we will screen patients ages 18 and older who have Medicaid, Medicare, or no health insurance, are able to read/write in English, and reside within a Philadelphia zip code. Patients will be excluded from participating in the research study if they are less than 18 years, deemed to be in critical condition by ED physicians, do not read/write in English, present in severe distress (e.g., respiratory, physical, or emotional), are under police custody, have primary commercial/private health insurance benefits, or do not have a stable mobile phone number for the next 21 days.

5. Early Withdrawal of Subjects:

Participation in this study involves the completion of a brief screening survey and a baseline survey that includes the BDT Benefits Launch Express (BLX) benefits eligibility survey prior to randomization. Participants who are not eligible for at least one of the benefits for which BDT provides application support will not be randomized to continue in the study but will receive compensation for the time spent completing the pre-randomization survey. Participants randomized to the intervention arm will receive an initial text message one-day post-discharge with instructions for connecting with BDT and a follow-up text message two days later on Day 3 to assess connection success. Participants will then receive subsequent reminders to connect with BDT on Days 7 and 14 post-discharge. The message schedule will be adjusted to ensure that text messages are received during weekday business hours, with 1-2 day shifts depending on the weekday of enrollment. Participants who indicate that they have successfully connected with BDT will not receive subsequent messages. Text messages to patients will leverage insights from behavioral economics to improve the response rate to call BDT (see attached message scripts).

All participants, both those randomized to the control and intervention groups, will receive a final study survey on Day 14 post-discharge to assess the participant's experiences in the research study as well as the contact phone number to reach the BDT enrollment counselors. All participants will be informed that they may withdraw consent and leave the study at any time for any reason. Participants will be informed that they may opt out of receiving study text messages at any time by texting the word 'BYE.' Participants will also be informed that they will still receive a final text message 14 days after discharge from the emergency department inviting them to complete the final online survey.

6. Vulnerable Populations:
N/A

7. Populations vulnerable to undue influence or coercion:
N/A

STUDY DESIGN:
We conducted a pilot study with 30 patients prior to launching the RCT in July 2023. The pilot study only enrolled participants to receive the intervention messages to test the design efficacy and implementation success of the text messaging system. We will now concurrently launch a two-arm prospective intervention randomized controlled trial that is expected to occur over 6 months in Penn Medicine Emergency Departments. The study will be conducted using Way to Health, a research information technology platform at the University of Pennsylvania used previously in digital health engagement clinical trials.

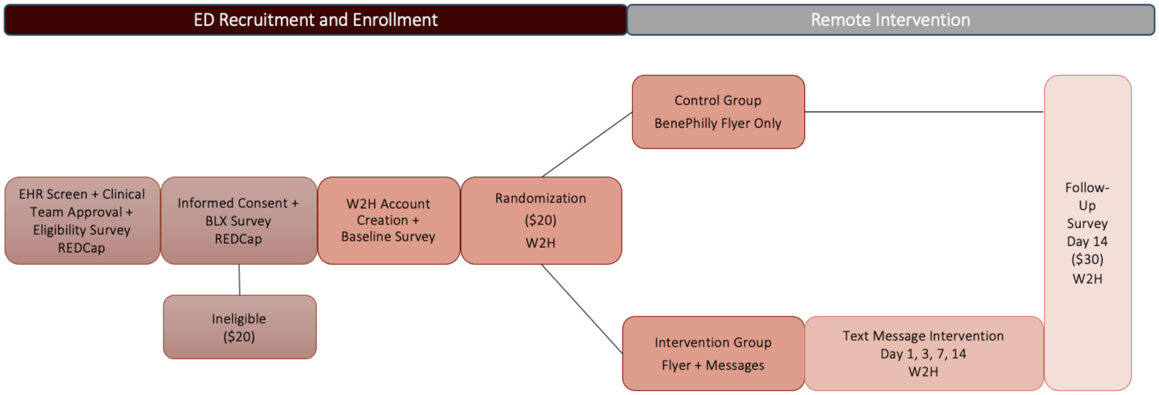

Figure 1. Participant Flowchart

The study team consisting of trained clinical research coordinators and Academic Associates, post-baccalaureate pre-health students employed in the ED to assist in clinical research activities, will recruit patients who are at least 18 years old who have Medicaid and/or Medicare health insurance, are able to read/write in English, and reside within a Philadelphia zip code. Patients will be excluded from participating in the research study if they are less than 18 years, deemed to be in critical condition by ED physicians and unlikely to be discharged, do not read/write in English, present in severe distress (e.g., respiratory, physical, or emotional), are under police custody, have primary private health insurance benefits or are designated self-pay or without health insurance, or do not have a stable mobile phone number for the next 21 days. Research staff will then approach patients and obtain informed consent using REDCap to confirm eligibility. The eligibility survey includes the BLX survey administered via secure website. In addition to meeting the study's inclusion/exclusion criteria, participants must also be eligible for at least one benefit for which BDT provides application assistance in order to be enrolled in the study and randomized. All participants will receive $20 for completing informed consent and the BLX survey. Eligible patients will then create an account through the study website on Way To Health, complete a brief baseline survey, and will then be randomized to either the intervention or control arms.

Active Control Arm

Participants in the control group will receive a summary flyer providing contact information for BDT, including the unique study phone line to connect with a BDT enrollment navigator. Currently, the standard of care is for ED patients to receive no information about public benefits. Therefore, the flyer represents an augmentation of usual care for patients randomized to the control group. Participants will also receive a one-time text message introduction to the study 2 hours after enrollment with a reminder that the study team will contact them in 14 days to complete a brief follow-up survey.

Intervention Arm

Participants in the intervention arm will be instructed that study personnel will be contacting them the first day after discharge to help connect them to a BDT enrollment navigator to help them submit their applications for public benefits. At Day 1 post-discharge, participants in the intervention group will receive a text message to prompt them to call the BDT enrollment navigators to complete their applications for public benefits they screened for while in the ED. On Day 3, patients will receive a follow-up message to assess whether they have reached out to BDT. Those participants who indicate they have not yet connected with BDT on Day 3 will receive reminder text messages at Day 7 and 14 post-discharge.

METHODS:

1. Study Instruments

Participants will complete three surveys as part of their study enrollment. The Eligibility Survey completed in REDCap, a HIPAA compliant platform, will confirm that participants meet the defined inclusion/exclusion criteria. The BLX survey, which is administered via a secure website, assesses participant's potential eligibility for public benefits and will be used to confirm participant eligibility in the study. Participants must be potentially eligible for at least one of the benefits programs for which BDT provides direct application or referral support. Results from the BLX survey will be manually entered into REDCap by trained clinical research personnel. Eligible patients will then create an account in Way To Health, a HIPAA compliant online research platform used for many clinical research studies across Penn Medicine entities, and complete a baseline survey collecting data related to participant sociodemographic characteristics, attitudes towards application for public benefits programs, and health-related social needs.

After 14 days, the follow-up survey will be administered in Way To Health. Participants in the control group will also receive a BDT Summary Flyer detailing the ways in which they may contact a BDT enrollment counselor to complete the benefits application process.

The Informed Consent, Eligibility Survey, and BLX Survey will be completed on REDCap. Participants will complete the Baseline survey and End of Study survey on the Way To Health platform. A demo version of the BLX survey can also be reviewed at https://uphs-staging.benefitslaunch.org/.

2. Group Modifications:

N/A

3. Method for Assigning Subjects to Groups:

Study participants will be randomized following informed consent, confirmation of eligbility, and completion of the baseline survey. Participants will be randomly assigned to either the control or intervention groups. Participants will be randomized in block sizes of 6 via Way To health.

4. Administration of Surveys and/or Process:

The online surveys will be collected in the Penn Medicine ED by trained research staff (clinical research assistants and/or Academic Associates). The eligibility surveys will take approximately 15 minutes to

complete via REDCap and the BLX survey website, and the baseline survey will take approximately 5-10 minutes to complete via Way To Health.

The Follow-Up survey will be accessed via text message and completed via Way To Health. The survey will take approximately 5-10 minutes to complete.

5. Data Management:

All study personnel that will use this data are listed on the IRB application and have completed training in HIPAA standards and the CITI human subjects research. Computer-based files will only be made available to personnel involved in the study through the use of access privileges and passwords. Wherever feasible, identifiers will be removed from study-related information. Precautions are in place to ensure the data are secure by using passwords and encryption because the research involves web-based surveys. The use of paper-based records will be limited. Paper-based records will be secured in a locked cabinet in the principal investigator's office, retained for a maximum of 7 years after study completion and then destroyed.

7. Subject Follow-up:

Participants will be contacted via text message to complete a brief survey via Way To Health of whether they were successfully enrolled in the benefits programs in which they had applied.

STUDY PROCEDURES:

1. Detailed Description:

Patients in Penn Medicine EDs will be recruited by trained clinical research personnel to participate in this two-arm prospective randomized controlled trial. Research personnel will pre-screen participants in the Electronic Health Record and confirm a positive likelihood of being discharged by the patient's clinical team (e.g., physicians or nurses). Research personnel will then approach participants, introduce the study, and ask the final pre-eligibility screening questions. Initially eligible and interested participants will provide electronic written informed consent and the BLX survey to confirm final eligibility. Participants must be eligible for at least one benefit for which BDT provides application assistance in order to continue in the study. Participants will then create an account in Way To Health and complete a baseline survey collecting information regarding sociodemographic characteristics, attitudes towards public benefits programs, and health-related social needs. All participants will receive $20 via Greenphire VISA ClinCard for completing informed consent and the BLX survey.

Participants will then be randomized to either the control or intervention groups. Participants in the control arm will then receive the BenePhilly Summary Flyer providing the contact information to connect with a BDT enrollment navigator. Control group participants will also receive a one-time introductory text two hours after enrollment reminding them that they will be contacted via text message in 14 days to complete a follow-up survey. Patients randomized to the intervention arm will receive the BenePhilly Summary Flyer and an initial text message two hours after enrollment with instructions to expect follow-up texts one day post-discharged from the ED. At Day 1 post-discharge from the ED, patients will receive their first text message to connect with benefits enrollment navigators at BDT to apply for the benefits they may be potentially eligible to receive. On Day 3 post-discharge, participants will receive a text message assessing whether they have yet connected to BDT. If participants indicate that they have not yet connected with BDT on day 3, they will receive subsequent reminder text messages to connect with BDT on Days 7 and 14 post-discharge. After 14 days post-discharge, all participants will receive a patient satisfaction survey asking about their experience in the study. Text message schedules were adjusted to ensure that patients receive messages during business hours when BDT call center was operational.

Eligible study participants will also be consented to allow the study team to extract relevant clinical data from the EHR, including listed co-morbidities, prior ED and hospital utilization, access to primary care,

and payor type. We will extract these patient-level variables using Epic Clarity, an automated programming interface which permits secure queries of the electronic health record.

All survey data in Penn Medicine EDs will be collected via REDCap and Way To Health on designated secure, password-protected tablets. The BLX survey portion of the Eligibility Survey will be completed via secure BDT website prepared for this research study.

2. Data Collection:

Survey data be collected using REDCap, Way To Health, and BDT. Data will also be collected via PennChart (EPIC) and patient self-report.

3. Genetic Testing: Not applicable

4. Use of Deception: Not applicable

STATISTICAL ANALYSIS PLAN

1. Detailed Description:

We will use descriptive statistics to summarize the data and patient characteristics. Baseline demographic and clinical characteristics will be reported as frequency and percent for categorical variables and median and distribution for continuous variables. We will compare baseline characteristics between intervention and control arms using t-tests for continuous variables and chi-squared tests for categorical variables. The goal of these comparisons will be to determine if the two arms are balanced on baseline variables after randomization.

Our primary analysis will focus on the difference in the number of phone calls received on the BDT phone line between the two arms. We will conduct an intent to treat (ITT) analysis. The ITT analysis will include all randomized participants in the groups to which they were randomly assigned. We will estimate and test differences in proportions between arms using two-sample z-tests. If necessary, multivariable logistic regression models that account for baseline measurements of patient demographic and clinical characteristics. Missing data will be assessed for patterns and multiple imputation will be used if deemed appropriate. A p-value of 0.05 will be deemed statistically significant but emphasis will be placed on point estimates and confidence intervals. Secondary outcomes including number of applications for public benefits submitted will also be compared using both unadjusted and adjusted models as described above.

2. Power Analysis:

The following provides a summary for the power analyses used to determine sample size and interim power analyses for the LEAP randomized controlled trial.

No Interim Analysis
Based on the literature and pilot work, we believe that the intervention (automated text messaging) can achieve a successful primary outcome (binary, whether or not a patient calls the benefits navigator) for 5-10% of study participants. We believe that the control group will achieve the same outcome 1% or less of the time. The minimum important difference that we believe is actionable for future interventions at scale is 5%. We elected to use one-sided testing given our primary interest in determining whether text messaging was more effective than paper flyers, which represented the standard of care. Given that this study had a limited funding and timing window, our power decisions also reflected practical considerations around study efficiency.

Percentage success in treatment group:	**10%**
Percentage success in control group:	1%
Alpha:	0.05
Beta:	0.20

Ratio of treatment to control:	1:1
One-sided test	
Total sample size required	158 participants

Interim Analysis

For practical reasons related to planning for study funding, timing of study completion, and allocation of research staff, we conducted one interim analysis. We did not specify specific stopping guidelines and planned to continue the study regardless of this interim analysis, but with potential changes in allocation of study personnel.

Alpha spending function using O'Brien Fleming efficacy boundary

For k = 2

P	0.0088	0.0467
N	80	160

Conclusion

Our target study enrollment is 160 total patients, with one pre-specified interim analysis (without pre-determined stopping guidelines or plan for study cessation) at approximately 50% enrollment.

3. Stata Commands:

gsdesign twoproportions 0.01 0.10, efficacy(obfleming) information (0.5 1) alpha(0.05) power(0.8) onesided
power twoproportions 0.01 0.15, alpha(0.0088) power(0.8) onesided.

RISK/BENEFIT ASSESSMENT:

1. Risks:

A potential risk to participants includes the risk of breach of confidentiality about any preferences and data shared in the survey; however, there are strong data safeguards in place to prevent confidentiality breaches.

2. Benefits:

The primary benefit of this project may be enrollment in public benefits programs. This study may also yield low-cost, generalizable knowledge to better understand how connecting to benefits programs may improve the health of populations presenting for care in Emergency Departments. Thus, the knowledge gained by this study may lead to significant improvements in enrollment for public benefits programs in the Philadelphia region and across the United States.

3. Subject Privacy:

Interested participants will be recruited directly in Penn Medicine EDs. Participants will be asked to complete an electronic written informed consent via REDCap. Informed consent will include a description of the voluntary nature of the participation, the study procedures, risks and potential benefits in detail. The enrollment procedure will provide the opportunity for potential participants to ask questions and review the consent form information with family and friends prior to making a decision to participate. Participants will be told that they do not have to answer any questions if they do not wish and can drop out of the study at any time, without affecting their medical care or the cost of their care. They will be told that they may or may not directly benefit from the study and that all information will be kept strictly confidential, except as

required by law. Participants will have access to a copy of the consent document. All efforts will be made by the study staff to ensure participant privacy.

All survey data will be stored in a secure, web-based database (Way to Health or REDCap) where a study ID number will be generated for each patient. A link between the study ID number and the patient PHI will need to be maintained to ensure that the study staff can track recruitment efforts to potential participants and to avoid contacting any patients who have previously declined to participate. To ensure that confidentiality is preserved, individual identifiers (such as name) are stored in a single password protected system that is accessible to study research, analysis and IT staff only. This system is hosted on site at UPenn and is protected by a secure firewall. Once a participant is in this system, they will be given a unique study ID number. Any datasets and computer files that leave the firewall will be stripped of all identifiers besides the study ID and individuals will be referred to by their study ID only, except when this information is required to share with Benefits Data Trust for collection of study outcomes (see below). The study ID will also be used on all analytical files. Way to Health and REDCap are secure web applications for building and managing online surveys and databases. Privacy of all study data will be maintained by restricting access to the identifiable information only to approved study staff who have received subject confidentiality and privacy training. Study coordinators will access patient contact information from the database to conduct recruitment phone calls. The study coordinator will review the consent script, which will include a description of the voluntary nature of participation, the study procedures, risks and potential benefits in detail. Participants will be told that all information will be kept strictly confidential, except as required by law. Subjects will be provided a copy of the consent document. All efforts will be made by study staff to ensure subject privacy. Enrollment will be conducted by the study coordinators who will enter patient information directly into the Way To Health platform once a participant has consented to participate. This database is hosted on a secure server as detailed in the subject confidentiality section. Study coordinators may have to contact patients in the intervention and their support partners during the course of the study and will use the WTH database to access contact information to facilitate this contact.

To collect outcomes data for whether patients called BDT and submitted applications for benefits, we will need to share a list of study participants with identifying information to BDT. We will provide BDT with a list of patients including participant first names, last names, and date of birth. BDT will then use this list to document whether these patients called BDT, on what dates, and the outcomes of the calls including benefits applied for. BDT will then share this completed dataset with the study team. BDT will be instructed to delete the transmitted file with patient information following confirmed return of the completed dataset. All data will be transferred using BDT's secure file transfer protocol (SFTP). SFTPs are folders that are accessed through an SFTP client (BDT uses FileZilla) that allows those with the log in information to access a secure, shared folder. Data can be easily passed back and forth, reducing concerns of data breaches. BDT will provide a guide for how to access the SFTP folder created for this study. Instructions containing log in information will be securely sent to the email provided to BDT. The following individuals will have access to the log in information to access shared data:

- Joseph Harrison, Project Manager
- Charles Rareshide, Senior Data Analyst

Further, BDT and the study team are executing a HIPAA Business Associates Agreement, which confirms BDT will comply with all aspects of HIPAA when exchanging the PHI of clients, including the Privacy and Security Rule. Additionally, BDT is certified as compliant with the American Institute of CPAs' System and Organization Controls (SOC2) in all five trust principles which include Confidentiality and Privacy as defined below.

· ***Confidentiality*** *addresses the company's ability to protect data that should be restricted to a specified set of persons or organizations. This includes client data intended only for company personnel, confidential company information such as business plans or intellectual property, or any other information required to be protected by law, regulations, contracts, or agreements*

· ***Privacy*** *criteria speaks to an organization's ability to safeguard personally identifiable information from unauthorized access. Personal information is collected, used, retained, disclosed, and disposed to meet entity's objectives*

4. Subject Confidentiality:

Patient information will be collected using REDCap and Way to Health on tablets used by the research personnel in the ED. The data to be collected include data on participant eligibility for public benefits programs and participant characteristics and sociodemographic information (e.g., age, gender, race, ethnicity, marital status, household income, number and type of dependents, etc.). Research material that is obtained will be used for research purposes only. The same procedure used for the analysis of automated data sources to ensure protection of patient information will be used for the survey data, in that patient identifiers will be used only for linkage purposes or to contact patients. The study identification number, and not other identifying information, will be used on all data collection instruments. All study staff will be reminded to appreciate the confidential nature of the data collected and contained in these databases. The Penn Medicine Academic Computing Services (PMACS) will be the hub for the hardware and database infrastructure that will support the project and is where the Way to Health web portal is based. The PMACS is a joint effort of the University of Pennsylvania's Abramson Cancer Center, the Cardiovascular Institute, the Department of Pathology, and the Leonard Davis Institute. The PMACS provides a secure computing environment for a large volume of highly sensitive data, including clinical, genetic, socioeconomic, and financial information. PMACS requires all users of data or applications on PMACS servers to complete a PMACS-hosted cyber-security awareness course annually, which stresses federal data security policies under data use agreements with the university. The curriculum includes Health Insurance Portability and Accountability Act (HIPAA) training and covers secure data transfer, passwords, computer security habits and knowledge of what constitutes misuse or inappropriate use of the server. We will implement multiple, redundant protective measures to guarantee the privacy and security of the participant data. All investigators and research staff with direct access to the identifiable data will be required to undergo annual responsible conduct of research, cybersecurity, and HIPAA certification in accordance with the University of Pennsylvania regulations. All data for this project will be stored on the secure/firewalled servers of the PMACS Data Center, in data files that will be protected by multiple password layers. These data servers are maintained in a guarded facility behind locked doors, with very limited physical access rights. They are also cyber-protected by extensive firewalls and multiple layers of communication encryption. Electronic access rights are carefully controlled by the University of Pennsylvania system managers. In addition, each participant will be assigned a unique identifier without identifying information, and data will be entered into an electronic database using only the unique identifier. Only trained study staff will have access to the code that links the unique identifier to the subject's identity.

Data will be stored, managed, and analyzed on a secure, encrypted server behind the University of Pennsylvania Health System (UPHS) firewall. All study personnel that will use this data are listed on the IRB application and have completed training in HIPAA standards and the CITI human subjects research. Data access will be password protected. Data will be completely deidentified following data collection and during the analysis and summary of results.

How will confidentiality of data be maintained? Check all that apply.

X Paper-based records will be kept in a secure location and only be accessible to personnel involved in the study.

X Computer-based files will only be made available to personnel involved in the study through the use of access privileges and passwords.

X Prior to access to any study-related information, personnel will be required to sign statements agreeing to protect the security and confidentiality of identifiable information.

X Whenever feasible, identifiers will be removed from study-related information.

X Precautions are in place to ensure the data is secure by using passwords and encryption, because the research involves web-based surveys.

5. Protected Health Information

Protected health information that will be collected by the study team include:

- Penn Chart (EPIC) medical record numbers
- Date of Birth
- First Name and Last Name
- E-mail address
- Phone number

This information (i.e., medical record numbers) will be deleted following data extraction during the analysis and summary phase of this study.

6. Compensation:

All participants will receive $20 for completing the informed consent and BLX survey and $30 for completing the follow-up survey 14 days post-discharge from the ED. All study payments will be disbursed using Greenphire VISA ClinCard.

7. Data and Safety Monitoring:

The Principal Investigator will be responsible for monitoring the study. All participants will be given anticipatory guidance on when to seek medical attention. In addition, participants will be asked to report to the study team any injuries or medical care that they feel resulted from participation in the study. They can either call the study team or send an email. Clinical research personnel will call the participant to collect information regarding the issue and then the PI will review and determine whether it is ok to proceed, further investigation is needed, or the participant should stop the study.

8. Investigator's Risk/Benefit Assessment:

Given the minimal risk to participants and low costs of this study, and the potential benefits that this study and future research it will inform may offer to those same participants and members of their communities, we believe that the benefits of this investigation far outweigh the risks.

INFORMED CONSENT:

1. *Consent Process:*

Informed consent will be obtained from all participants at the beginning of their study participation prior to completing the eligibility survey. Upon recruitment, individuals who are interested in learning more about the study will review the study information including its objectives, duration, and requirements with research personnel. The informed consent will detail the voluntary nature of the study, the risks and benefits of participation, alternatives to participation, and that participants can withdraw from the study at any time. Participants will be asked to sign the informed consent document to agree to participate in the study. Participants will be provided with details regarding how to contact the research team via email or phone at any time if they subsequently wish to withdraw from the study.

Participants will be given contact information (including telephone and email) for the principal investigator (Dr. Kilaru), and the University of Pennsylvania IRB to address any potential concerns. A detailed Manual of Operations will be developed for the study and procedures will be designed to ensure efficient and prompt updating. All study participants read and review the informed consent document with assistance from in-person research assistants. All study staff will undergo a thorough training and certification procedure prior to protocol implementation, including CITI Course in the Protection of Human Research

Participants online training in research ethics. The initial training will be conducted by the PI and will include an overview of the study, detailed explanation of the study protocol, and hands-on practice of specific protocol components. All study staff will be observed conducting randomly selected protocol duties at least twice annually and evaluated using specifically designed checklists.

2. ***Waiver of Informed Consent:***

N/A
