# Supplementary material for: Linkage of Emergency Department Patients With Public Benefits Navigators via Text Messages: A Randomized Clinical Trial
Source: JAMA Health Forum. 2026 Feb 6;7(2):e256637. doi: 10.1001/jamahealthforum.2025.6637 (PMC12881984; doi:10.1001/jamahealthforum.2025.6637)
Supplement: Supplement 2. — eFigure 1. Study overview diagram eTable 1. List of public benefits with application support offered by benefits navigators from Benefits Data Trust eTable 2. Schedule of text messages according to weekday of study enrollment eFigure 2. Automated text messages used in the intervention arm eFigure 3. Flyer distributed to control and intervention groups eMethods 1. Baseline survey eMethods 2. Power analysis eTable 3. Frequency of discontinued and failed text messages in the intervention group eTable 4. Baseline survey—health-related social needs eTable 5. Baseline survey—previous experience with public benefits applications eTable 6. Final survey responses eTable 7. Sensitivity analysis (adjusted model) [file jamahealthforum-e256637-s002.pdf]

# Supplemental Online Content

Kilaru AS, Haider A, Harrison J, et al. Linkage of emergency department patients with public benefits navigators via text messages: a randomized clinical trial. *JAMA Health Forum*. 2026;7(2):e256637. doi:10.1001/jamahealthforum.2025.6637

**eFigure 1.** Study overview diagram
**eTable 1.** List of public benefits with application support offered by benefits navigators from Benefits Data Trust
**eTable 2.** Schedule of text messages according to weekday of study enrollment
**eFigure 2.** Automated text messages used in the intervention arm
**eFigure 3.** Flyer distributed to control and intervention groups
**eMethods 1.** Baseline survey
**eMethods 2.** Power analysis
**eTable 3.** Frequency of discontinued and failed text messages in the intervention group
**eTable 4.** Baseline survey—health-related social needs
**eTable 5.** Baseline survey—previous experience with public benefits applications
**eTable 6.** Final survey responses
**eTable 7.** Sensitivity analysis (adjusted model)

This supplemental material has been provided by the authors to give readers additional information about their work.

© 2026 Kilaru AS et al. *JAMA Health Forum*.

**eFigure 1**
Study Diagram

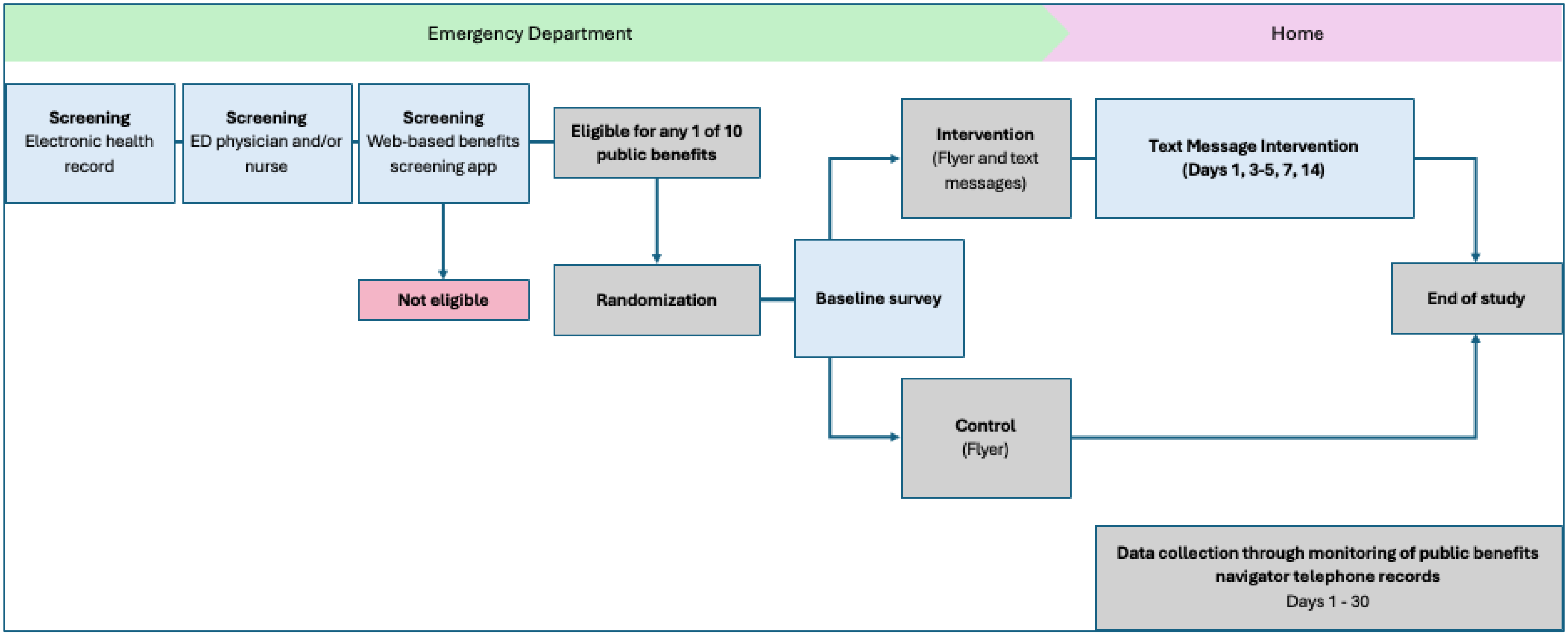


© 2026 Kilaru AS et al. *JAMA Health Forum*.

**eTable 1**
List of public benefits with application support offered by benefits navigators

| Public benefits with option to directly submit application by navigator | | |
|---|---|---|
| **Benefit** | **Type / Source** | **Description** |
| Low Income Home Energy Assistance Program (LIHEAP) | Federal | Income-based cash grants program to help Pennsylvania families pay heating bills and other weatherization needs |
| Property Tax & Rent Rebate Program (PTRR) | State | Income-based program providing rent rebates ranging from $380 to $1,000 to eligible older adults and people with disabilities who are homeowners or renters |
| Children's Health Insurance Program (CHIP) | State / Federal | Health insurance program that covers uninsured children up to age 19 who are not eligible for Medicaid, at either no cost or low cost for families |
| Supplemental Nutrition Assistance Program (SNAP) | Federal | Food benefits for low-income families to supplement grocery budget, administered through an Electronic Benefits Transfer (EBT) ACCESS Card to make food purchases |
| Child Care Income Subsidy (CCIS) | State | Subsidized child-care program helping low-income families pay child-care fees with income- and work-based eligibility requirements |
| Homestead exemption | Municipal | Property tax reduction for owner-occupied homes serving as a primary residence |
| Senior Food Box Program | Federal | Supplemental food packages for low-income older adults |
| Pharmaceutical Assistance Contract for the Elderly (PACE) Program | State | Program to lower out-of-pocket costs for medications for individuals at least 65 years of age |
| Medicare Savings Programs (MSP) | State | Income and asset-eligible program to help pay Medicare out-of-pocket costs |
| Low Income Subsidy (LIS) | Federal | Medicare program to help people with limited income and resources pay drug coverage premiums, deductibles, coinsurance, and other costs |
| **Public benefits with option for application support** | | |
| Child tax credit (CTC) | Federal | Federal tax credits and rebates for families with qualifying child dependents |
| Earned income tax credit (EITC) | Federal | Federal tax credits and rebates for low- and moderate-income workers and families |
| Free Application for Federal Student Aid (FAFSA) | Federal | Application to determine eligibility for grants, scholarships, work-study programs, and educational loans |

© 2026 Kilaru AS et al. *JAMA Health Forum*.

| | | |
|---|---|---|
| Medicaid | State / Federal | Government-based health insurance for adults and children with limited income and resources |
| Health Insurance Exchange | Federal | Online marketplace where individuals can compare and apply for commercial health insurance plans |
| Social Security Disability Insurance (SSDI) | Federal | Monthly payments to people who have a disability that stops or limits their ability to work. |
| Supplemental Security Income (SSI) | Federal | Monthly payments to people with disabilities and older adults who have little or no income or resources |
| Temporary Assistance for Needy Families (TANF) | Federal | Time-limited financial assistance for pregnant individuals and families with dependent children |
| Unemployment insurance | State / Federal | Cash benefits to eligible workers after a no-fault loss of employment |
| Women, Infants, and Children (WIC) | State / Federal | Supplemental food, nutrition education, and healthcare referrals for low-income pregnant, postpartum, and breastfeeding women, infants and children (up to age 5) |

© 2026 Kilaru AS et al. *JAMA Health Forum*.

**eTable 2**
Schedule of text messages according to weekday of study enrollment

Text messages were intended to be sent between 10:30am and 1pm, during weekday when the benefits navigator call center was open. Study recruitment occurred on weekdays, between 9am and 5pm.

To accommodate the availability of the benefits navigator call center, the text message schedule was adjusted to skip weekend days (and holidays, although not depicted in this table).

On Mondays and Tuesdays, the text messages were sent according to the expected schedule (Day 1, 3, 7, 14). On Wednesdays, the Day 3 message was delayed until Day 5. On Thursdays, the Day 3 message was delayed until Day 4. On Fridays, the Day 1 message was delayed until Day 3 and the Day 3 message was delayed until Day 5, to preserve equivalent spacing between text messages.

| | Mon | Tues | Weds | Thurs | Fri | Sat | Sun | Mon | Tues | Weds | Thurs | Fri |
|---|---|---|---|---|---|---|---|---|---|---|---|---|
| Mon | 0 | 1 | | 3 | | | | 7 | | | | |
| Tues | | 0 | 1 | | 3 | | | | 7 | | | |
| Weds | | | 0 | 1 | | | | 5 | | 7 | | |
| Thurs | | | | 0 | 1 | | | 4 | | | 7 | |
| Fri | | | | | 0 | | | 3 | | 5 | | 7 |

© 2026 Kilaru AS et al. *JAMA Health Forum*.

**eFigure 2**
Automated text messages

***Legend***
*Example screenshots of text messages sent to study participants in the intervention group: Messages on day 1 and day 3 were adjusted to next business day if occurring on weekend. See* ***Supplement*** *for flyer distributed to both intervention and comparison groups.*

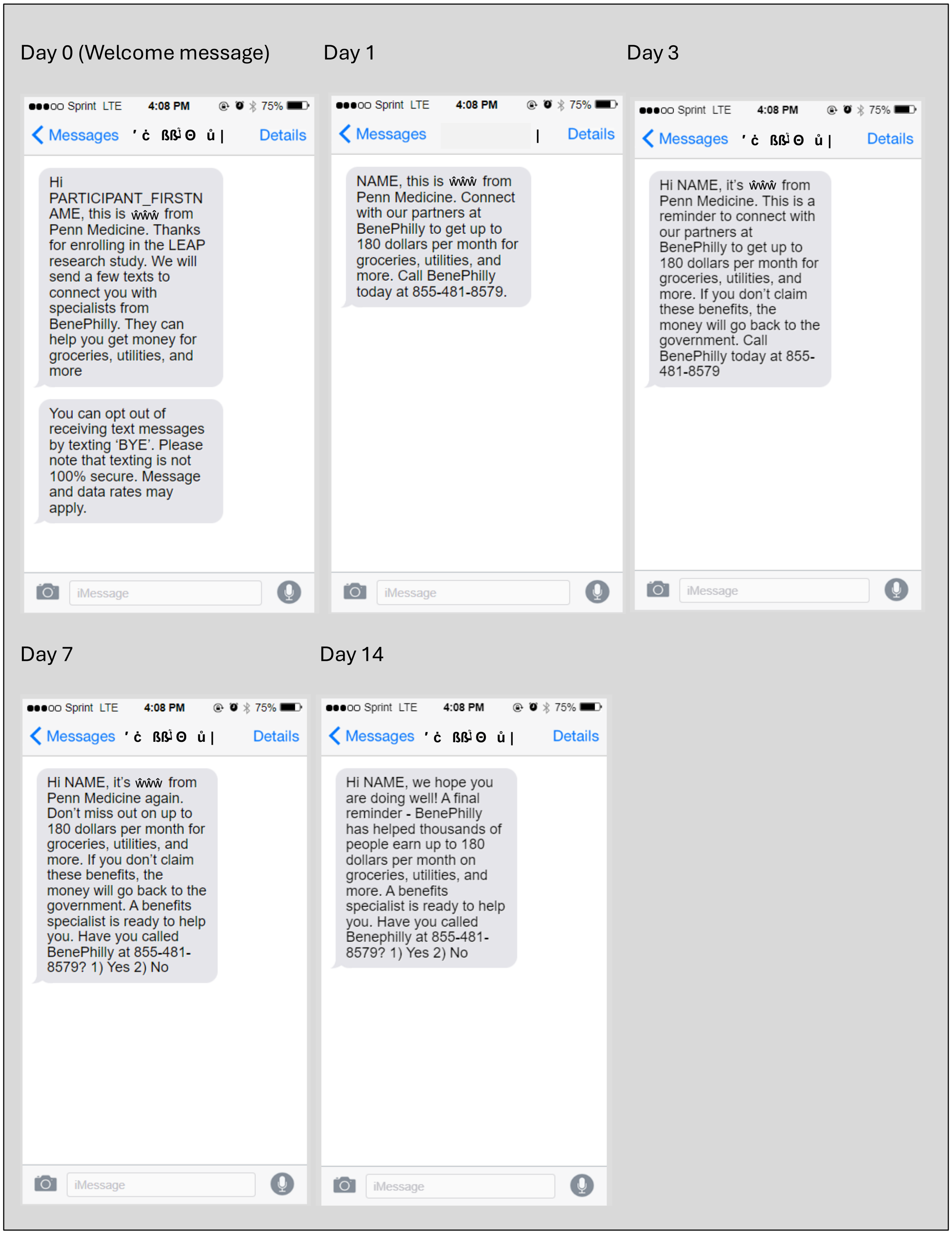


© 2026 Kilaru AS et al. *JAMA Health Forum*.

**eFigure 3**
Flyer distributed to both control and intervention groups

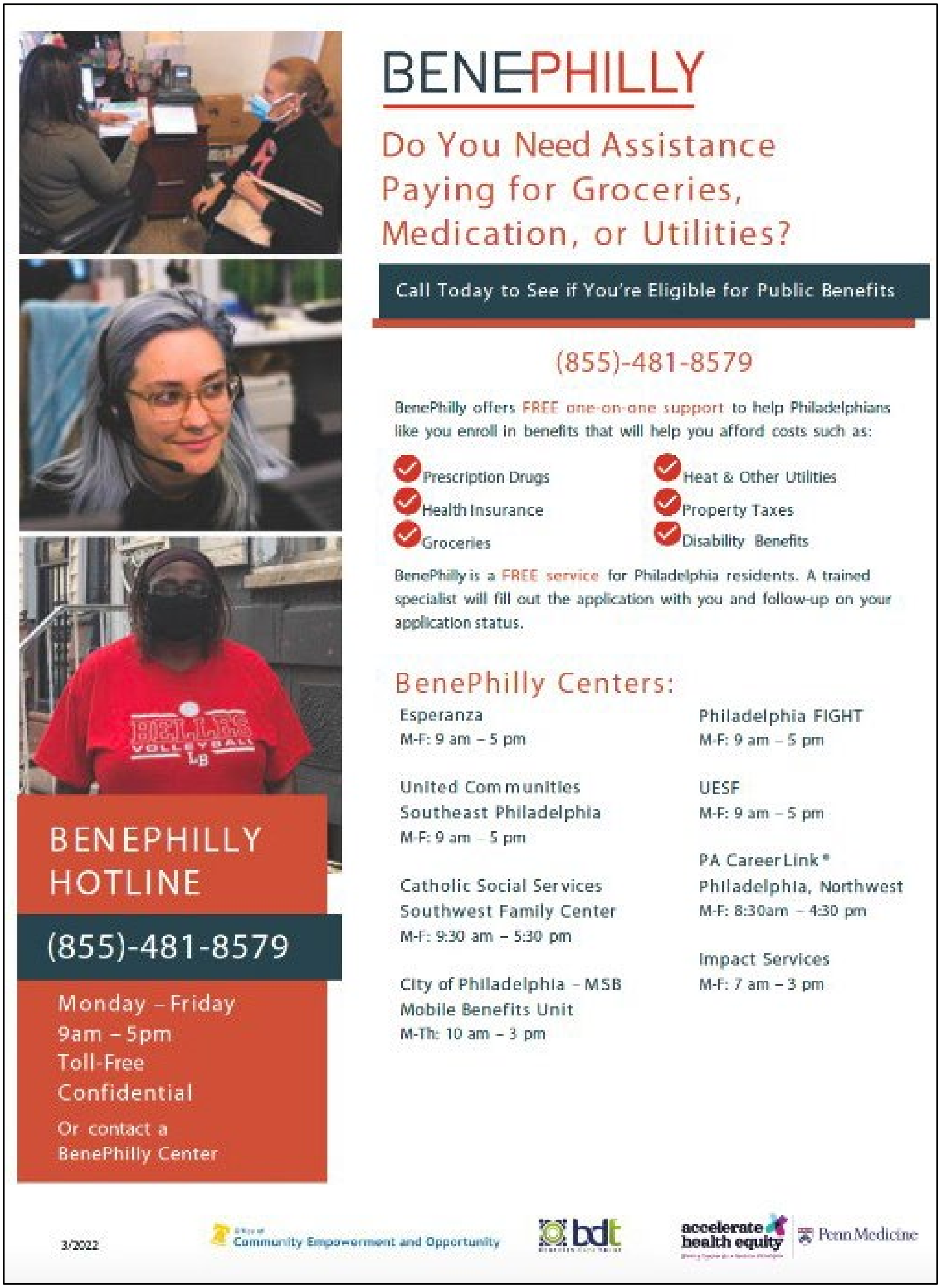


© 2026 Kilaru AS et al. *JAMA Health Forum*.

**eMethods 1**
Baseline survey

**Participant Characteristics**

1. How would you describe your gender?
   - o Male (1)
   - o Female (2)
   - o Transgender/trans man (3)
   - o Transgender/trans woman (4)
   - o Genderqueer/gender nonconforming neither exclusively male nor female (5)
   - o Decline to answer (6)
   - o Other (7)

2. How would you describe your sexual orientation?
   - o Straight or heterosexual (1)
   - o Lesbian or gay (2)
   - o Bisexual (3)
   - o Queer, pansexual, and/or questioning (4)
   - o I don't know (5)
   - o Decline to answer (6)
   - o Other (7)

3. What is the highest level of education you have completed?
   - o Less than high school degree (1)
   - o High school graduate (high school diploma or equivalent including GED) (2)
   - o Some college but no degree (3)
   - o Associate degree in college (2-year) (4)
   - o Bachelor's degree in college (4-year) (5)
   - o Master's degree (6)
   - o Doctoral degree (7)
   - o Professional degree (JD, MD) (8)
   - o Decline to answer (9)

4. What is your total yearly **household** income before taxes?
   - o Less than $10,000 (1)
   - o $10,000 - $19,999 (2)
   - o $20,000 - $29,999 (3)
   - o $30,000 - $39,999 (4)
   - o $40,000 - $49,999 (5)
   - o $50,000 - $59,999 (6)
   - o $60,000 - $69,999 (7)
   - o $70,000 - $79,999 (8)
   - o $80,000 - $89,999 (9)
   - o $90,000 - $99,999 (10)
   - o $100,000 - $149,999 (11)
   - o More than $150,000 (12)
   - o Decline to answer (13)

**5.** How many people live in your household with you?

**Previous Experiences with Benefits**

© 2026 Kilaru AS et al. *JAMA Health Forum*.

1. Have you applied for benefit programs in the past?
    - o Yes (1)
    - o No (0)
    - o Decline to answer (2)

2. (IF YES TO #10): On a scale of 1 to 5, how difficult was it to apply for benefits programs?
    - o Number Selection 1 to 5
    - o Decline to answer (6)

3. (IF YES TO #10): Which of the following have been challenges for you in applying for benefits programs in the past?
    - ☐ I was unsure of how to apply (1)
    - ☐ The process for applying took too long (2)
    - ☐ I was unable to collect the necessary paperwork (3)
    - ☐ I did not have a permanent address (4)
    - ☐ I didn't want to give information to the government (5)
    - ☐ I found it too confusing or overwhelming (6)
    - ☐ I was concerned about what others would think about me (7)
    - ☐ I am not a person who would ever apply for government benefits (8)
    - ☐ Other: _____ (9)

4. (IF NO TO #10): On a scale of 1 to 5, how difficult do you think it would be to apply for benefits programs?
    - o Number Selection 1 to 5
    - o Decline to answer (0)

5. (IF NO TO #10): Which of the following have prevented you from applying for benefits programs in the past?
    - o I was unsure of how to apply (1)
    - o The process for applying took too long (2)
    - o I was unable to collect the necessary paperwork (3)
    - o I did not have a permanent address (4)
    - o I didn't want to give information to the government (5)
    - o I found it too confusing or overwhelming (6)
    - o I was concerned about what others would think about me (7)
    - o I am not a person who would ever apply for government benefits (8)
    - o Other: _____ (9)

6. On a scale of 1 to 5, how confident do you feel to navigate the benefits application process?
    - o Number Selection 1 to 5
    - o Decline to answer (0)

**<u>Health-Related Social Needs</u>**

1. In the past 12 months, to what extent are you worried about having a safe and healthy place to live?
    - o Number 1 to 5 (not worried at all to very worried)
    - o I do not have a steady place to live. (6)
    - o Decline to answer (7)

2. In the past 12 months, to what extent have you been worried that your food would run out before you got money to buy more?

© 2026 Kilaru AS et al. *JAMA Health Forum*.

- Number 1 to 5 (not worried at all to very worried)
- I have run out of food. (6)
- Decline to answer (7)

3. In the past 12 months, to what extent have you been worried that a lack of reliable transportation will keep you from medical appointments, meetings, work, or from getting things needed for daily living?
   - Number 1 to 5 (not worried at all to very worried)
   - A lack of transportation has already interfered with my daily living. (6)
   - Decline to answer (7)

4. In the past 12 months, to what extent have you worried about the electric, gas, oil, or water company shutting off services in your home?
   - Number 1 to 5 (not worried at all to very worried)
   - Already shut off (6)
   - Decline to answer (7)

5. On a scale of 1 to 5, how difficult is it for you to pay for the very basics like food, housing, medical care, and heating?
   - Number Selection 1 to 5
   - I cannot afford basic necessities. (0)
   - Decline to answer (9)

6. In the past 12 months, to what extent did you have strong family and community support in your everyday life?
   - Number Selection 1 to 5
   - I do not have family and/or community support. (0)

Over the past 2 weeks, how often have you been bothered by any of the following problems?

7. Little interest or pleasure in doing things?
   - Not at all (0)
   - Several Days (1)
   - More than half the days (2)
   - Nearly every day (3)

8. Feeling down, depressed, or hopeless?
   - Not at all (0)
   - Several Days (1)
   - More than half the days (2)
   - Nearly every day (3)

© 2026 Kilaru AS et al. *JAMA Health Forum*.

**eMethods 2.**
Power analysis

**Power Analysis**
The following provides a summary for the power analyses used to determine sample size and interim power analyses for the LEAP randomized controlled trial.

***No Interim Analysis***
Based on the literature and pilot work, we believe that the intervention (automated text messaging) can achieve a successful primary outcome (binary, whether or not a patient calls the benefits navigator) for 5-10% of study participants. We believe that the control group will achieve the same outcome 1% or less of the time. The minimum important difference that we believe is actionable for future interventions at scale is 5%. We elected to use one-sided testing given our primary interest in determining whether text messaging was more effective than paper flyers, which represented the standard of care. Given that this study had a limited funding and timing window, our power decisions also reflected practical considerations around study efficiency.

| | |
|---|---|
| Percentage success in treatment group: | **10%** |
| Percentage success in control group: | 1% |
| Alpha: | 0.05 |
| Beta: | 0.20 |
| Ratio of treatment to control: | 1:1 |
| One-sided test | |
| | |
| Total sample size required | 158 participants |

**Interim Analysis**
For practical reasons related to planning for study funding, timing of study completion, and allocation of research staff, we conducted one interim analysis. We did not specify specific stopping guidelines and planned to continue the study regardless of this interim analysis, but with potential changes in allocation of study personnel.

Alpha spending function using O'Brien Fleming efficacy boundary

| For k = 2 | | |
|---|---|---|
| *P* | 0.0088 | 0.0467 |
| N | 80 | 160 |

**Conclusion**
Our target study enrollment is 160 total patients, with one pre-specified interim analysis (without pre-determined stopping guidelines or plan for study cessation) at approximately 50% enrollment.

**Stata Commands**
gsdesign twoproportions 0.01 0.10, efficacy(obfleming) information (0.5 1) alpha(0.05) power(0.8) onesided
power twoproportions 0.01 0.15, alpha(0.0088) power(0.8) onesided

© 2026 Kilaru AS et al. *JAMA Health Forum*.

**eTable 3**
Frequency of discontinued and failed text messages in the intervention group

| | **Welcome** | **Day 1** | **Day 3** | **Day 7** | **Day 14** |
|---|---|---|---|---|---|
| **Text message error**, n (%) | 0 (0%) | 0 (0%) | 4 (5%) | 8 (10%) | 4 (5%) |
| **Text message discontinuation,** n (%) | 0 (0%) | 0 (0%) | 0 (0%) | 1 (1%) | 1 (1%) |

© 2026 Kilaru AS et al. *JAMA Health Forum*.

**eTable 4**
Baseline survey – health-related social needs

| **Health-related social needs*** | | **All patients** N = 160 | **Intervention** N = 79 | **Control** N = 81 |
|---|---|---|---|---|
| **Housing security** | **Mean score** | **1.9** | **1.9** | **1.9** |
| | No steady place to live | 5 (3%) | 2 (3%) | 3 (4%) |
| | Decline to answer | 0 (0%) | 0 (0%) | 0 (0%) |
| **Food security** | **Mean score** | **2.3** | **2.3** | **2.3** |
| | Run out of food | 2 (1%) | 2 (3%) | 0 (0%) |
| | Decline to answer | 1 (1%) | 0 (0%) | 1 (1%) |
| **Transportation** | **Mean score** | **2.2** | **2.1** | **2.3** |
| | Already lack transportation | 3 (2%) | 3 (4%) | 0 (0%) |
| | Decline to answer | 0 (0%) | 0 (0%) | 0 (0%) |
| **Utilities** | **Mean score** | **1.9** | **1.9** | **2.0** |
| | Already shut off | 1 (1%) | 1 (1%) | 0 (0%) |
| | Decline to answer | 3 (2%) | 1 (1%) | 2 (2%) |
| **Financial strain** | **Mean score** | **2.3** | **2.3** | **2.4** |
| | Decline to answer | 1 (1%) | 1 (1%) | 0 (0%) |
| **Family and community support** | **Mean score** | **3.7** | **3.8** | **3.5** |
| | Decline to answer | 3 (2%) | 2 (3%) | 1 (1%) |
| **PHQ2** | 0-2 | 116 (73%) | 61 (77%) | 55 (68%) |
| | 3-6 | 44 (28%) | 18 (23%) | 26 (32%) |

*Social needs survey questions, with the exception of PHQ2, were asked in format of 5-point Likert scale, with mean score calculated as weighted average of responses. Participants were given option to respond outside of 5-point responses if the question did not apply; see Supplement S6 for survey questions as administered.

© 2026 Kilaru AS et al. *JAMA Health Forum*.

**eTable 5**
Baseline survey – previous experience with public benefits applications

| | | All patients N = 160 | Intervention N = 79 | Control N = 81 |
|---|---|---|---|---|
| **Confidence in navigating benefits applications*** | Mean score | 3.8 | 3.9 | 3.8 |
| | Decline to answer | 48 (30%) | 27 (34%) | 21 (26%) |
| **Previous applications for benefits** | Yes | 114 (71%) | 53 (67%) | 61 (75%) |
| | No | 43 (27%) | 23 (29%) | 20 (25%) |
| | Decline to answer | 3 (2%) | 3 (4%) | 0 (0%) |
| ***If yes to previous applications for benefits*** | | | | |
| **Challenges with prior applications** | | ***N = 114*** | ***N = 53*** | ***N = 61*** |
| | Unsure how to apply | 20 (18%) | 8 (15%) | 12 (20%) |
| | Application took too long | 27 (24%) | 11 (21%) | 16 (26%) |
| | Unable to collect paperwork | 19 (17%) | 5 (9%) | 14 (23%) |
| | No permanent address | 13 (11%) | 6 (11%) | 7 (11%) |
| | Didn't want to give information to government | 3 (3%) | 1 (2%) | 2 (3%) |
| | Too confusing or overwhelming | 13 (11%) | 3 (6%) | 10 (16%) |
| | Concerned about what others would think | 3 (3%) | 1 (2%) | 2 (3%) |
| | Not a person who would ever apply for benefits | 5 (4%) | 1 (2%) | 4 (7%) |
| **Ease of application*** | Mean score | 2.5 | 2.3 | 2.6 |
| | Decline to answer | 46 (33%) | 26 (33%) | 20 (25%) |
| ***If no to previous applications for benefits*** | | | | |
| **Barriers to applying for benefits** | | ***N = 43*** | ***N = 23*** | ***N = 20*** |

© 2026 Kilaru AS et al. *JAMA Health Forum*.

| | | | | |
|---|---|---|---|---|
| | Unsure how to apply | 19 (44%) | 12 (52%) | 7 (35%) |
| | Application took too long | 3 (7%) | 0 (0%) | 3 (15%) |
| | Unable to collect paperwork | 4 (9%) | 2 (9%) | 2 (10%) |
| | No permanent address | 5 (12%) | 3 (13%) | 2 (10%) |
| | Didn't want to give information to government | 2 (5%) | 1 (4%) | 1 (5%) |
| | Too confusing or overwhelming | 6 (14%) | 3 (13%) | 3 (15%) |
| | Concerned about what others would think | 0 (0%) | 0 (0%) | 0 (0%) |
| | Not a person who would ever apply for benefits | 2 (5%) | 1 (4%) | 1 (5%) |

*Questions asked as part of 5-point Likert scale, with mean score calculated as weighted average of responses. See Supplement S6 for survey questions as administered.

© 2026 Kilaru AS et al. *JAMA Health Forum*.

**eTable 6**
Final survey responses

| | | All patients<br>N = 118 | Intervention<br>N = 60 | Control<br>N = 58 |
|---|---|---|---|---|
| **Confidence in navigating benefits applications*** | Mean score | 3.5 | 3.5 | 3.6 |
| | Decline to answer | 4 (3%) | 3 (5%) | 1 (2%) |
| **Ease of application*** | Mean score | 2.6 | 2.3 | 2.7 |
| | Decline to answer | 11 (9%) | 9 (15%) | 2 (3%) |
| **To what extent did text messages make you want to call BenePhilly?*** | | | | |
| | Mean score | | 4.0 | n/a |
| | Decline to aswer | | 1 (2%) | n/a |
| **How much did you like the text message reminders?*** | | | | |
| | Mean score | | 4.0 | n/a |
| | Decline to answer | | 2 (4%) | n/a |
| **Could a team member call you to learn more about your experiences?** | | | | |
| | Yes | 88 (75%) | 44 (77%) | 44 (73%) |
| | No | 30 (25%) | 16 (23%) | 14 (27%) |
| *Questions asked as part of 5-point Likert scale, with mean score calculated as weighted average of responses. | | | | |

© 2026 Kilaru AS et al. *JAMA Health Forum*.

**eTable 7**
Sensitivity analysis, adjusting for baseline benefits eligibility

| | **Intervention** N = 79 | | **Control** N = 81 | |
|---|---|---|---|---|
| | Unadjusted percentage | Adjusted probability (95% CI) | Unadjusted percentage | Adjusted probability (95% CI) |
| **Call to benefits navigator within 14 days** | 25% | 28% (19 to 38) | 0% | 1% (-1 to 4) |
| **Call to benefits navigator within 30 days** | 30% | 32% (22 to 42) | 2% | 4% (0.05 to 8) |
| **Any benefits application submitted within 14 days** | 14% | 17% (9 to 25) | 0% | 1% (-2 to 4) |
| **Any benefits application submitted within 30 days** | 18% | 20% (11 to 28) | 0% | 1% (-2 to 4) |

The adjusted model accounts for whether participants had previous applications for public benefits (Table 1) and public benefits eligibility determined by initial screening (Table 2), for the following benefits: LIHEAP, PTRR, CHIP, SNAP, CCIS, Homestead Exemption, PACE, MSP, LIS.

The multivariable logistic regression model uses Firth's penalized likelihood approach to account for the rarity of outcomes, specifically the zero events in the control group for the primary outcome. Analytic code and de-identified study data is available from the authors upon request.

© 2026 Kilaru AS et al. *JAMA Health Forum*.
