# Supplementary material for: Linkage of Emergency Department Patients With Public Benefits Navigators via Text Messages: A Randomized Clinical Trial
Source: JAMA Health Forum. 2026 Feb 6;7(2):e256637. doi: 10.1001/jamahealthforum.2025.6637 (PMC12881984; doi:10.1001/jamahealthforum.2025.6637)
Supplement: Supplement 3. — Data sharing statement [file jamahealthforum-e256637-s003.pdf]

Data Sharing Statement

Kilaru. Linkage of Emergency Department Patients With Public Benefits Navigators via Text Messages. *JAMA Health Forum*. Published February 06, 2026.
doi:10.1001/jamahealthforum.2025.6637

Data
Additional Information: Linking Emergency Department Patients to Assistance Programs Study (LEAP), https://clinicaltrials.gov/study/NCT05654220, NCT 05654220
Data available: Yes
Data types: Deidentified participant data, Data dictionary
How to access data: austin.kilaru@pennmedicine.upenn.edu
When available: With publication

Supporting Documents
Document types: Statistical/analytic code, Informed consent form
How to access documents: austin.kilaru@pennmedicine.upenn.edu
When available: With publication

Additional Information
Who can access the data: Researchers whose proposed use of the data has been approved
Types of analyses: For any purpose
Mechanisms of data availability: After approval of a proposal
